# Parental presence is associated with parent–child agreement in quality of life assessment among 5- to 7-year-old children: a comparison of administration conditions using the Dutch PedsQL

**DOI:** 10.1007/s11136-026-04200-4

**Published:** 2026-06-06

**Authors:** E. E. Hartman, W. H. M. Emons, F. J. Oort

**Affiliations:** 1 Center of Research on Psychological Disorders and Somatic Diseases (CoRPS), Department of Medical and Clinical Psychology, Tilburg University, Warandelaan 2, 5037 AB Tilburg, The Netherlands; 2 Department of Methodology and Statistics, Tilburg University, Tilburg, the Netherlands; 3 Research Institute of Child Development and Education, Faculty of Social and Behavioral Sciences, University of Amsterdam, Amsterdam, The Netherlands

**Keywords:** Quality of life, Young children, Self-report, Parent-proxy report, Administration mode

## Abstract

**Purpose:**

The Pediatric Quality of Life Inventory™ (PedsQL™ 4.0) is widely used to assess quality of life (QoL) in children, yet evidence on the reliability and validity of young children’s self-reports is inconsistent. We evaluated whether self-reported QoL in young children varies by parental presence during administration and whether parent–child agreement differed between mothers and fathers.

**Methods:**

Secondary analyses were conducted using data from primary schools (*n* = 303, children aged 5–7 years) including at least one participating parent. Children completed the PedsQL self-report either at school with a trained research assistant (parent-absent) or at home with a parent who read items aloud and recorded answers (parent-present). Mothers and fathers completed parallel proxy-reports. Multilevel modeling was used to estimate mean differences and correlations between reporters and conditions, with age and sex as covariates.

**Results:**

Internal consistency of child self-reports was limited across the four subdomains, with somewhat lower values in the parent-absent condition. Parent ratings showed no systematic differences between conditions, whereas children scored higher when a parent was present, yielding smaller parent–child gaps and higher correlations. These patterns were similar for mothers and fathers.

**Conclusions:**

In this school-based community sample, improved agreement with a parent present was driven by higher child scores, consistent with brief, non-leading parental assistance (clarification/recall). Self-reports of young children obtained without a parent present warrant caution. Clear, age-appropriate guidance on administration and structured parental support is needed.

**Supplementary Information:**

The online version contains supplementary material available at 10.1007/s11136-026-04200-4.

## Plain English summary

We looked at whether children aged 5–7 can give reliable answers about their own quality of life, that is, how they feel about their health, emotions, friends, school and play, and whether having a parent present makes a difference in how they answer. We worked with 303 children from regular primary schools. Each child completed the same short questionnaire either (a) at school with a trained assistant and no parent present, or (b) at home with a parent who read the questions aloud and noted the child’s answers. Mothers and fathers also filled in a matching questionnaire about their child. In general, parents tended to rate their child’s QoL higher than the children rated themselves. Parents’ ratings were about the same whether or not they were with the child. Children, however, scored higher with a parent present. As a result, the difference between parent and child scores was smaller when a parent provided support. Mothers and fathers showed very similar patterns. This means that little, non-leading support from a parent, such as reading the question in simple words or helping the child remember examples, can help young children express their own view more clearly while keeping the child’s voice central. By contrast, results collected without a parent present should be interpreted with extra care. Clear, age-appropriate guidance on how parents can support would help make these assessments more consistent and fair.

## Introduction

The combined use of child self‑reports and parent proxy‑reports is considered best practice for assessing quality of life (QoL), particularly in younger age groups where self‑reports may be less reliable [1, 2]. Over recent decades, QoL assessment in children has received increasing attention, reflecting its growing importance as a key outcome in both research and applied settings [3–5]. QoL is generally conceptualized as a multidimensional, subjective construct encompassing physical, emotional, social, and school functioning [6].

However, there is ongoing debate about the age at which children can provide a reliable and valid self‑report of their QoL. Most instruments set the lower age limit at eight years, assuming that younger children lack the necessary cognitive and linguistic skills [2, 7, 8]. Nevertheless, American studies using the Pediatric Quality of Life Inventory (PedsQL™) have shown that children as young as five can provide meaningful self‑reports [9, 10], particularly when supported by simplified response formats and parental assistance [3, 11–13].

In contrast, international studies using translated versions of the PedsQL have shown mixed psychometric performance, with lower internal consistency in younger children reported in Dutch and other non-U.S. samples [14–17], and parent-parent agreement varied across domains and instruments (e.g., PedsQL vs. CHU9D) [18]. A plausible explanation is methodological, particularly differences in administration procedures. Varni’s team instructed parents to assist children during questionnaire completion [3, 11–13], whereas many other studies used independent, interviewer-administered formats without parental involvement 

[14–17]. Conijn et al. (2020) reported low internal consistency for several PedsQL subscales and only modest parent–child agreement in 4- to 8-year-olds [19], but did not test whether child self-report quality varied by administration mode (parent-present vs. parent-absent) and relied solely on mother proxies, reflecting a broader tendency to prioritize maternal perspectives. Indeed, most prior studies have used mothers almost exclusively as proxy respondents [13, 20], consistent with traditional caregiving roles [21, 22]. In clinical populations, mothers often rate children’s QoL lower than the children do, particularly on psychosocial domains [23, 24], and agreement is typically higher for mother–child than father–child dyads [25–27]. By contrast, in community samples only one study has explicitly compared fathers and mothers. It found low-to-moderate parent–child agreement with no meaningful mother–father differences [28].

We evaluated internal consistency and parent–child agreement for the Dutch PedsQL in young children (ages 5–7 years), comparing a school-based administration with parents absent and a home-based administration with parents present. Agreement was quantified using mixed-effects (multilevel) modeling to estimate mean differences and correlations between child self-reports and parent proxy-reports (mothers and fathers).

## Methods

### Participants and procedure

This secondary analysis used data from a large Dutch study on parent–child agreement in QoL among a community-based sample, collected between 2008 and 2011 through a network of primary schools in the southeastern region of the Netherlands. The study complied with the Declaration of Helsinki (2000 revision) and was considered outside the scope of the Dutch Medical Research Involving Human Subjects Act (WMO) as it was a non-medical study. A subset of these data was previously reported by Conijn et al. (2020) [19], which included children aged 4–8 years and only mother proxy-reports in the parent-present condition. The current study takes a different approach by comparing parent-present and parent-absent administration and by distinguishing between mother and father reports. For the present analysis, we included only families with children aged 5–7 years recruited in 2010–2011, when data from both fathers and mothers were available. The children were required to have basic proficiency in Dutch.

Trained psychology students coordinated school contact and questionnaire administration. Assignment of administration mode for child self-report (school-based with parents absent vs. home-based with parents present) was made in consultation with each school, based on practical considerations, and allocated at the classroom level.

In the parent-present condition, both the child self-report and the parent proxy-reports were sent home with an information letter and informed consent form. Children completed the self-report with the assistance of one parent, who read items aloud and recorded the child’s answers. Parents were instructed to complete the proxy form before assisting with the self-report. Forms were returned in sealed envelopes via classroom collection boxes. In the parent-absent condition, children completed the self-report at school with a trained psychology student, while parents completed the proxy-reports at home and returned them via the same procedure. Only questionnaires accompanied by signed informed consent were included. Parents also completed a brief demographic form covering child sex(boy/girl) and age (5, 6, or 7 years). Exact participation rates could not be calculated because the number of invited families was not consistently recorded.

### Measures

#### Generic quality of life

Generic quality of life was assessed with the PedsQL™ 4.0 Generic Core Scales [9], using the Dutch translation by Bastiaansen and Koot [14]. Permission for use was obtained from the original authors. The PedsQL consists of 23 items covering four domains: Physical (8 items), Emotional (5 items), Social (5 items), and School (5 items) functioning. Items refer to problems experienced during the past month. Both child self-reports and parent proxy-reports from mothers and fathers were collected using parallel versions of the questionnaire with different response scales. For the proxy-report, items were rated on a 5-point Likert scale ranging from 0 (“never a problem”) to 4 (“almost always a problem”). For the child self-report (ages 5–7), a simplified 3-point response scale was used (“never,” “sometimes,” “almost always”), each accompanied by smiley-face symbols [10]. In both administration conditions, the adult (parent or research assistant) read the items aloud and recorded the child’s response without providing interpretation or guidance. As prescribed in the PedsQL scoring manual, all items were reverse scored and linearly transformed to a 0–100 scale, with higher scores indicating better QoL. For the parent proxy-report (5-point scale), response options were transformed as follows: 0 = 100, 1 = 75, 2 = 50, 3 = 25, 4 = 0. For the child self-report (3-point scale), responses were coded 0, 2, and 4 and transformed as follows: 0 = 100, 2 = 50, and 4 = 0. Subscale scores were computed by averaging the transformed item scores, and a Psychosocial Summary Score was derived by averaging the Emotional, Social, and School Functioning subscales. Missing data were handled according to the 50% rule [29]. The PedsQL has demonstrated acceptable psychometric properties for self-reports in U.S. samples of children aged 5 –7 years, with internal consistency coefficients (α) ranging from 0.68 to 0.73 for most subscales and somewhat lower values for School Functioning (α = 0.59 to 0.63) [3, 9]. In the Netherlands, however, previous research has shown limited internal consistency for self-reports at ages 6 and 7 (Physical: α = 0.44; Emotional: α = 0.43; Social: α = 0.63; School: α = 0.40) [14, 15]. In the Dutch normative study by Engelen et al. (2009), parent proxy-reports for 5- to 7-year-olds demonstrated acceptable internal consistency across most PedsQL subscales, supporting the use of proxy-reports in this age group [30].

### Statistical analyses

Analyses were conducted in SPSS (v28). Statistical significance was set at 5% (p < .05, two-sided). Internal consistency coefficients α were computed in R (v4.5.0) using the psych package.

#### Sample and study characteristics

To compare the two administration conditions (parent-present vs. parent-absent), an independent-samples t-test was used to compare child age, and χ² tests were conducted for parent availability, child sex, and age group. The PedsQL domain scores (Emotional, Social, School, Physical, and the Psychosocial Summary Score) are presented in Supplementary Table S1 for context. The analytic sample included children with an available self-report and at least one parent proxy-report (n = 303), comprising 277 mother reports and 268 father reports.

#### Internal consistency analyses

To assess internal consistency across administration conditions, α values were computed separately by domain and reporter (child, mother, father), stratified by condition (parent-present vs. parent-absent). α coefficients and 95% confidence intervals (CIs) were obtained using 1,000 bootstrap resamples. Following conventional guidelines for group-level research, we considered α ≥ .70 indicative of acceptable internal consistency, and values < .70 were interpreted as limited [31]. Differences in internal consistency between administration conditions were considered meaningful only when 95% CIs did not overlap, a conservative criterion for reliable differences.

#### Agreement across administration conditions

To obtain maximum likelihood estimates (MLE) of regression effects (representing mean differences between reports) and correlations (between reports) [32], multilevel analyses were conducted using the SPSS MIXED procedure, accounting for dependencies between assessments (level 1) within families (level 2). The advantage of this approach is that it uses all available information, including information from families with one missing parent report. The PedsQL domains (Emotional, Social, School, Physical) and the Psychosocial Summary Score served as dependent variables. Child age and sex (0 = boy, 1 = girl) were included as covariates.

#### Mean differences by condition

To compare mean QoL between child self-reports and parent proxy-reports and to examine differences by administration condition and parent role, reporter (child, mother, father), condition (parent-present vs. parent-absent), and their interaction (reporter × condition) were entered as fixed effects in the multilevel model. Two equivalent parameterizations were used for interpretability. In the first model, the intercept represents the mean self-report of boys when parents were absent. In the second model, the intercept represents the mean self-report of boys when parents were present.

As all dependent variables were rescaled to a mean of 0 and a standard deviation of 1, the regression coefficients can be interpreted as effect sizes (d), with values of 0.2, 0.5, and 0.8 indicating small, medium, and large effects, respectively [33].

#### Correlations

To further examine the relative agreement between parents and children, multilevel models were run separately by administration condition (parent-absent vs. parent-present) and stratified by parent (mother, father) to estimate correlations between child self-reports and parent proxy-reports. Differences in correlations between conditions were considered meaningful if the 95% CIs did not overlap.

## Results

### Sample and study characteristics

Table 1 shows the distribution of parent availability, child sex, and age by administration condition. A total of 303 children participated, with 197 (65.0%) in the parent-present condition and 106 (35.0%) in the parent-absent condition. Every child provided a self-report and at least one parent (mother or father) returned a proxy-report with sufficient item completion to compute PedsQL domains. Consequently, all children were eligible for the agreement analyses (mother n = 277; father n = 268). Most children had reports from both parents (n = 242, 79.9%), 35 (11.6%) had only a mother report, and 26 (8.6%) had only a father report. Parent availability differed by condition: in the parent-present condition, 192 children (97.5%) had a mother report and 174 (88.3%) had a father report, whereas in the parent-absent condition, 85 children (80.2%) had a mother report and 94 (88.7%) had a father report. The difference in parent availability was significant, χ^2^(2) = 26.44, p < .001.

**Table 1:** Parent availability, child sex and age distribution in the administration condition

Characteristics (*N* = 303)	Parent-present (home) (*n* = 197)	Parent-absent (school) (*n* = 106)	*p*
*Parent availability, n (%)*			< .001*
Mother, no father	23 (11.7)	12 (11.3)	
Father, no mother	5 (2.5)	21 (19.8)	
Both parents	169 (85.8)	73 (68.9)	
*Child sex, n (%)*			.880
Boys	91 (46.2)	48 (45.3)	
Girls	106 (53.8)	58 (54.7)	
*Child age, n (%)*			
5 years	48 (24.4)	37 (34.9)	.074
6 years	71 (36.0)	39 (36.8)	
7 years	78 (39.6)	30 (28.3)	
Mean age (*M*, *SD*)	6.15 (0.79)	5.93 (0.80)	.023*

*p < .05

With respect to child sex, the proportions of boys and girls did not differ between conditions, χ^2^(1) = 0.02, p = .880. Children in the parent-absent condition were on average slightly younger (mean (M) = 5.93 years, standard deviation (SD) = 0.80) than those in the parent-present condition (M = 6.15, SD = 0.79), although the magnitude of this difference was small, *t*(301) = 2.29, *p* = .023. Consistent with this, the parent-absent condition included a higher proportion of 5-year-olds (34.9%) compared to the parent-present condition (24.4%), and fewer 7-year-olds (28.3% vs. 39.6%), although the overall distribution across the three age groups did not reach statistical significance, χ^2^(2) = 5.20, p = .074.

### Reliability of self- versus proxy-reports across conditions

As shown in Table 2, internal consistency of child self-reports remained below the conventional threshold (α ≥ .70) [31] across all four subdomains. In the parent-absent condition, values ranged from 0.33 (Emotional) to 0.53 (School). In the parent-present condition values ranged from 0.51 (Social/School) to 0.60 (Emotional) . Although internal consistency coefficients tended to be higher in the parent-present condition, the conservative criterion of non-overlapping 95% confidence intervals did not provide evidence for meaningful differences in reliability between conditions. Across both conditions, parent proxy-reports showed satisfactory internal consistency in all domains.

**Table 2:** Internal consistency coefficients α (95% CI) for PedsQL self- and proxy-reports by administration condition

Reporter	Child		Mother		Father	
	Parent-present (*n* = 197)	Parent-absent (*n* = 106)	Parent-present (*n* = 192)	Parent-absent (*n* = 85)	Parent-present (*n* = 174)	Parent-absent (*n* = 94)
PedsQL	*α* (95% CI)	*α* (95% CI)	*α* (95% CI)	*α* (95% CI)	*α* (95% CI)	α (95% CI)
Emotional	.60 (.51–.68)	.33 (.06–.53)	.78 (.72–.83)	.72 (.60–.80)	.75 (.67–.81)	.68 (.55–.77)
Social	.51 (.38–.62)	.44 (.26–.59)	.81 (.75–.86)	.82 (.74–.87)	.81 (.74–.86)	.73 (.62–.82)
School	.51 (.38–.60)	.53 (.34–.69)	.72 (.64–.78)	.69 (.60–.76)	.80 (.70–.87)	.70 (.59–.78)
Psychosocial^1^	.74 (.69–.79)	.64 (.53–.73)	.86 (.82–.88)	.86 (.81–.90)	.86 (.83–.88)	.84 (.79–.87)
Physical	.54 (.43–.66)	.41 (.23–.58)	.75 (.66–.85)	.77 (.64–.89)	.78 (.69–.87)	.61 (.46–.76)

Internal consistency was evaluated using a conservative rule of thumb based on non-overlapping 95% confidence intervals. No differences in internal consistency between conditions met this criterion

^1^Psychosocial Summary Score = mean of Emotional, Social, and School Functioning (PedsQL)

### Agreement across administration conditions

Parent–child agreement was evaluated using group-level mean differences and correlations (in multilevel models), presented in Tables 3–4, respectively. PedsQL descriptives appear in Supplementary Table S1.

**Table 3:** Parameter estimates from multilevel models of quality of life domains by reporter and administration condition

	Emotional	Social	School	Psychosocial^1^	Physical
	*β* (*SE*), *p*	*β* (*SE*), *p*	*β* (*SE*), *p*	*β* (*SE*), *p*	*β* (*SE*), *p*
*Panel A — parameterization 1 (using absence as reference)*					
Child self-report (absent, boy)	− 0.18 (0.12), *p* = .140	− 0.86 (0.11), *p* < .001	− 1.05 (0.11), *p* < .001	− 0.89 (0.11), *p* < .001	− 0.85 (0.09), *p < .001*
Child: parent present vs absent (present vs absent)	0.21 (0.14), *p* = .113	0.70 (0.12), *p* < .001	1.02 (0.13),* p* < .001	0.82 (0.12), *p* < .001	0.82 (0.12), *p* < .001
Mother vs child (absent)	0.08 (0.12), *p* = .531	0.92 (0.11), *p* < .001	1.17 (0.11), *p* < .001	0.92 (0.11), *p* < .001	1.05 (0.12), *p* < .001
Father vs child (absent)	0.06 (0.12), *p* = .587	1.04 (0.12), *p* < .001	1.10 (0.11), *p* < .001	0.94 (0.10), *p* < .001	1.19 (0.11), *p* < .001
*Panel B — parameterization 2 (using presence as reference)*					
Child self-report (present, boy)	0.04 (0.09), *p* = .682	− 0.16 (0.08), *p* = .061	− 0.04 (0.09), p = .675	− 0.07 (0.09), *p* = .440	− 0.03 (0.08), *p* = .743
Mother vs child (present)	− 0.05 (0.09), *p* = .605	0.36 (0.08), *p* < .001	0.11 (0.08), *p* = .149	0.18 (0.08), *p* = .017	0.13 (0.08), *p* = .117
Father vs child (present)	− 0.03 (0.09), *p* = .768	0.41 (0.08), *p* < .001	0.10 (0.08), *p* = .232	0.21 (0.08), *p* = .008	0.21 (0.08), *p* = .010
*In both parameterizations*					
Child sex (girl vs boy)	0.04 (0.09), *p* = .640	0.04 (0.08), *p* = .665	0.17 (0.08), *p* = .044	0.10 (0.09),* p* = .238	− 0.03 (0.08), *p* = .762
Child age (*z*)	− 0.04 (0.05), *p* = .388	− 0.03 (0.04), *p* = .424	− 0.02 (0.04), *p* = .577	− 0.04 (0.04), *p* = .355	0.07 (0.04), *p* = .085

Panel A (parameterization 1, reference = boys’ self-reports in the parent-absent condition) and Panel B (parameterization 2, reference = boys’ self-reports in the parent-present condition) present two parameterizations of the same multilevel model. Because the reference group differs, intercepts change, but the underlying parent–child differences and condition contrasts are identical in magnitude (with sign reversed where applicable). ‘Mother/Father vs child (absent/present)’ are within-condition parent–child differences (parent−child). ‘Child: parent present vs absent’ is the contrast in child self-reports between administration conditions. Child sex (boy = 0, girl = 1) and child age (*z*) were included as covariates in all models; their coefficients are shown only in Panel A because they are identical across parameterizations. All continuous predictors were z-standardized. Bold indicates *p* < .05.

^1^Psychosocial Summary Score = mean of Emotional, Social, and School Functioning (PedsQL)

**Table 4:** Correlations between child self-reports and parent proxy-reports (mothers, fathers) across administration conditions

PedsQL	MotherChildC–		Father–Child	
	Parent-present (*n* = 192)	Parent-absent (*n* = 85)	Parent-present (*n* = 174)	Parent-absent (*n* = 94)
	*r* (95% CI)	*r* (95% CI)	*r* (95% CI)	*r* (95% CI)
Emotional	0.47 (0.36–0.58)*	− 0.04 (− 0.27–0.19)*	0.48 (0.36–0.58)*	0.06 (− 0.14–0.26)*
Social	0.44 (0.32–0.54)*	− 0.08 (− 0.29–0.13)*	0.31 (0.17–0.44)*	− 0.13 (− 0.32–0.07)*
School	0.54 (0.43–0.63)*	− 0.13 (− 0.35–0.10)*	0.50 (0.38–0.60)*	− 0.03 (− 0.24–0.19)*
Psychosocial^1^	0.54 (0.43–0.63)*	− 0.11 (− 0.32–0.10)*	0.55 (0.44–0.64)*	− 0.10 (− 0.29–0.09)*
Physical	0.40 (0.28–0.52)*	− 0.11 (− 0.34–0.12)*	0.44 (0.32–0.55)*	− 0.23 (− 0.42 to − 0.03)*

Asterisk (*) indicates a meaningful difference between the two administration conditions within the same dyad (mother-child or father-child) based on non-overlapping 95% confidence intervals (CIs) of the correlations. Correlations and 95% CIs were estimated from multilevel models with free within-family covariances, adjusted for child sex and age

^1^Psychosocial Summary Score = mean of Emotional, Social, and School Functioning (PedsQL)

#### Mean differences, adjusted for child sex and age

*Model and parameterizations*. Table 3 presents parameter estimates from multilevel models predicting each PedsQL domain. For interpretability, we report the same model with two parameterizations, presented in two separate panels. In parameterization 1 (Panel A), the parent-absent condition is the reference group, whereas in parameterization 2 (Panel B), the parent-present condition is the reference group. In both parameterizations, the intercept reflects boys’ self-reported QoL at the sample-mean age, mother and father coefficients represent deviations from child self-report within each condition, and child sex and standardized age (z-score) were included as covariates.

*Child by condition*. Children reported higher QoL with a parent present (vs. absent) on Social (*β* = 0.70), School (*β* = 1.02), Psychosocial (*β* = 0.82), and Physical (*β* = 0.82), but remained lower than parent ratings in both conditions.

*Parent by condition*. Parents rated higher than the child in both conditions, with no consistent difference in mean parent ratings between the parent-present and parent-absent condition; only small domain-specific shifts emerged (mothers − 0.10 to + 0.14 *SD*; fathers − 0.16 to + 0.12 *SD*; see Table 3, Panels A–B).

*Parent–child gap by condition*. In the parent-absent condition, parent–child gaps were large (= 0.9–1.2 SD, all *p* < .001 for Social, School, Psychosocial, and Physical), reflecting the higher parent ratings relative to children’s scores. In the parent-present condition (Panel B), these gaps were markedly smaller and remained significant only for Social (mother *β* = 0.36; father *β* = 0.41; both *p* < .001), Psychosocial (mother *β* = 0.18, *p* = .017; father *β* = 0.21, *p* = .008), and fathers’ ratings for Physical (*β* = 0.21, *p* = .010). *Covariate* effects were minimal: girls scored slightly higher on School than boys (*β* = 0.17, *p* = .044), and no other age or sex effects emerged.

### Correlations, adjusted for child sex and age

Table 4 shows correlations between child self-reports and parent proxy-reports (mothers and fathers) by administration condition. Parent–child agreement was consistently higher when a parent was present, with moderate correlations across domains for mother–child (*r* = 0.40 to 0.54) and father–child (*r* = 0.31 to 0.55) dyads. When parents were absent, correlations were near zero (*r* = − 0.13 to 0.06), and for Physical Functioning in the father-absent condition the association was even moderately negative (*r* =  − 0.23, 95% CI [− 0.42, − 0.03]), indicating inverse father–child rankings on that domain. Using a conservative rule of thumb of nonoverlapping 95% CIs across conditions, within-dyad comparisons showed meaningful differences across all QoL domains, favoring child–parent dyads in the present condition.

## Discussion

This study examined whether Dutch children aged 5–7 years can provide sufficiently interpretable self-reports on the PedsQL, and whether having a parent present during administration affects response quality. Overall, the internal consistency of child self-reports was limited and was lower when children completed the questionnaire at school without a parent present. When a parent was present, parent–child discrepancies were smaller and agreement was higher. Importantly, parents’ own ratings were broadly similar across conditions, whereas children scored higher with a parent present, which likely contributed to the smaller gaps. These results indicate that the administration context matters for how young children understand and answer QoL questions.

Together, our results indicate that having a parent present may help young children understand and complete the questionnaire, which may enhance the interpretability of their self-reports. Coefficients α were lower in our sample than those typically reported in U.S.-based studies [3, 11–13], even with a parent present, whereas the level of parent–child agreement in the parent-present condition was comparable to that reported in Varni’s studies. This pattern aligns with earlier discrepancies between non-U.S. and U.S. studies that may reflect differences in administration mode rather than problems with the measure itself. Comparable patterns have been reported in Dutch, Portuguese, and Japanese PedsQL validation studies, with somewhat lower internal coefficients in younger or clinically referred samples, while parent–child agreement is generally in the acceptable range [14–17].

Because administration procedures and instructions were identical across conditions, the higher parent–child agreement with a parent present likely reflects informal, neutrality-preserving assistance (e.g., clarifying item wording, aiding recall) [3, 11–13, 34, 35]. At the group-mean level, parent scores showed no systematic differences between conditions, whereas children scored higher when a parent was present, reducing the average parent–child gap. At the individual (relative) level, parent–child correlations increased from near zero in the parent-absent condition (*r* =  − 0.23 to 0.06) to moderate in the parent-present condition (mother–child *r* = 0.40–0.54; father–child *r* = 0.31–0.55), indicating greater alignment within parent–child dyads. Taken together, these results suggest that administration procedures can shift children’s mean scores and improve parent–child agreement, likely because parental assistance helps with item interpretation and supports recall. This interpretation aligns with evidence that children often struggle to grasp the intended meaning of health dimensions and response options [35]. Accordingly, parent-absent administrations should be interpreted with caution. Where feasible, brief structured guidance for parents (neutral paraphrasing, timeframe guidance, non-leading memory prompts) can enhance comparability without steering responses. Importantly, although agreement improved with parental presence, the internal consistency of child self-reports remained limited, underscoring that higher concordance does not, by itself, establish greater validity. For practical guidance on child HRQoL survey administration and age-appropriate procedures, we point readers to the Australian QUOKKA Research Program on child HRQoL methods [36].

Beyond agreement, common psychometric indices, such as internal consistency and parent–child correlations, may be incomplete or even misleading in young children [19]. Response styles that are typical at this age (e.g., extreme or invariant responding, limited differentiation across opposites) can distort internal consistency estimates and make them difficult to interpret, because they may primarily capture momentary experiences rather than stable functioning [37, 38]. Parents also do not necessarily represent children’s internal states, so higher agreement does not automatically imply better construct validity [34, 39]. Moreover, children’s self-reports at this age may reflect immediate, situational experiences rather than stable functioning over the past month, as intended by the PedsQL questionnaires. One illustrative example came from a 5-year-old participant, Johanna (pseudonym), who reported on the PedsQL that she had difficulty walking, yet had no difficulty running. She explained this by saying that she had tripped earlier that day in the schoolyard. This response pattern is consistent with the tendency of young children to prioritize immediate, situational experiences over trait-like judgments [40–42]. It remains unclear whether parental presence truly improves construct validity. Discrepancies may also arise from developmental limitations in comprehension or item interpretation [34], as well as from response biases that are typical of young children [43].

This interpretation aligns with prior theoretical and empirical work suggesting that shared completion can enhance agreement by aligning contextual frames of reference [34, 35], and is consistent with the rationale behind the structured dyadic interview approach described by Ungar et al. [44, 45]. Although our study did not include an interviewer-facilitated format, the results suggest that even limited parental involvement, such as reading items aloud or prompting recall, may support comprehension and item interpretation. 

Beyond child-related factors, we examined whether mother versus father ratings influenced reliability or agreement. In this community sample, maternal and paternal proxy reports were highly similar, and we found no meaningful differences in their agreement with child self-reports, suggesting that either parent may be able to provide support during assessment. These findings align with Hijkoop et al. (2021), who likewise reported comparable agreement for father–child and mother–child dyads in a community sample [28], but contrast with clinical studies in which agreement is typically higher for mothers than for fathers [25–27].

## Strengths and limitations

This study adds to the literature by focusing on a clearly defined age group in a community sample of 5–7-year-olds and by comparing two administration formats. The design allowed a direct comparison between self-reports completed with a parent present and those completed at school with a trained psychology student. Although instructions were identical in both conditions and restricted the adult’s role to reading items aloud and recording responses, this naturalistic contrast provides insight into how administration context can influence the interpretability of QoL self-reports in young children. A further strength is the inclusion of both mothers and fathers as proxy informants, allowing a balanced test of differences between mothers and fathers that are rarely examined in non-clinical samples.

We also combined group-level comparisons of means with individual-level correlations to assess parent–child agreement. In the psychosocial domains of the parent-present condition, parents and children showed both small mean differences and high correspondence in their scores. This alignment would have been missed if only means or only correlations had been considered. Using both indices provides a more robust and nuanced view of parent–child agreement [46, 47].

Nonetheless, several limitations should be noted. Generalizability may be limited by selection bias in a school-based community cohort from the southeastern region of the Netherlands. The administration groups were unequal in size, and children in the parent-absent condition were on average slightly younger than those in the parent-present condition. Age was controlled in all analyses, but residual confounding cannot be ruled out. No demographic or other characteristics of participating parents were collected, limiting insight into factors that might influence proxy reporting. Father reports were also less frequently available in the parent-present condition, which may have introduced additional imbalance.

Given the regional sample, unequal group sizes, slight age differences, missing parent characteristics, and fewer father reports in the parent-present condition, caution is warranted in generalizing these results. Moreover, the data were collected between 2010 and 2011. While the constructs remain relevant, shifts in societal norms and parenting practices (e.g., increased digital exposure, evolving parent–child communication) may influence how parents support children during assessments [48]. Replication in larger and more diverse cohorts is therefore needed, especially since only one prior study has examined proxy-reports from both fathers and mothers- in a general population of 5–7-year-olds [28], and studies that explicitly compare administration modes remain scarce.

Independent completion of mother and father proxy forms could not be verified in the home setting. For context, mother–father correlations were moderate (*r* = 0.50–0.68 across domains), which may partly reflect shared context or communication. Such dependence could inflate parent–parent agreement and modestly attenuate parent–child gaps. Our multilevel models, however, estimated mother–child and father–child effects separately. Still, some dependence between parental reports remains a plausible source of bias.

## Implications for practice

In this study, the adult’s role was limited to reading items aloud and recording responses. The higher agreement in the parent-present condition suggests that some parents may have provided informal scaffolding (e.g., neutral clarification, recall support). This calls in to question the assumption that strict adult neutrality is always preferable and points to the value of guided, neutrality-preserving assistance. We recommend that, in large-scale or routine assessments, parents receive brief, structured instructions (neutral paraphrasing of items, helping the child consider an appropriate timeframe, non-leading memory prompts) and that any assistance be documented. Providing such structured support may improve the comparability and interpretability of young children’s self-reports while maintaining the child’s perspective [35].

Despite longstanding emphasis on the child’s perspective, major organizations (e.g., FDA, ISPOR, NIHR, WHO) still provide no age-specific guidance on when and how to use self- and proxy-reports in young children [49]. Our findings are consistent with the emphasis on the child's perspective, but underscore a policy gap: clear, age-specific recommendations are needed regarding administration procedures and the appropriate role of structured parental assistance when collecting self- and proxy-reports from young children.

## Conclusion

This study refines understanding of reliability and parent–child agreement in a school-based sample of young children (5–7 years) using multilevel models that include reporter (child, mother, father), administration condition, and their interactions. Having parent and child complete the questionnaire together may enhance aspects of response quality while preserving the child’s perspective. Higher agreement in the parent-present condition likely reflects subtle, informal support by parents, such as clarifying wording or aiding recall, even without explicit instruction. These results support the development of age-appropriate guidelines for QoL assessment in early school age and call for transparent reporting of who was present and how items were delivered. Future studies should test standardized, developmentally informed protocols across settings to balance support with autonomy and ensure comparability.

## Supplementary Information

Below is the link to the electronic supplementary material.

Supplementary Material 1
