# Supplementary material for: Parental presence is associated with parent–child agreement in quality of life assessment among 5- to 7-year-old children: a comparison of administration conditions using the Dutch PedsQL
Source: Qual Life Res. 2026 Jun 6;35(7):186. doi: 10.1007/s11136-026-04200-4 (PMC13242433; doi:10.1007/s11136-026-04200-4)
Supplement: Supplementary file 1 — Supplementary Material 1 [file 11136_2026_4200_MOESM1_ESM.docx]

**Supplement**

**Table S1.**

Descriptive statistics for PedsQL scale scores by reporter and administration condition

| **PedsQL** | **Child self-report** | | **Mother report** | | **Father report** | |
| --- | --- | --- | --- | --- | --- | --- |
|  | **Present** | **Absent** | **Present** | **Absent** | **Present** | **Absent** |
|  | (*n* = 197)  *M (SD)* | (*n* = 106)  *M (SD)* | (*n* = 192)  *M (SD)* | (*n* = 85)  *M (SD)* | (*n* = 174)  *M (SD)* | (*n* = 94)  *M (SD)* |
| **Emotional** | 77.3 (17.5) | 74.2 (17.2) | 76.7 (15.3) | 76.4 (14.2) | 77.2 (14.4) | 75.2 (12.9) |
| **Social** | 80.0 (14.6) | 69.0 (18.5) | 85.8 (14.7) | 83.4 (14.6) | 86.6 (14.8) | 86.1 (12.4) |
| **School** | 83.2 (14.8) | 67.1 (19.5) | 84.9 (13.7) | 85.8 (12.3) | 84.9 (15.4) | 84.9 (12.5) |
| **Psychosocial^1^** | 80.2 (12.2) | 70.1 (13.5) | 82.4 (11.7) | 81.8 (11.4) | 82.9 (11.6) | 82.1 (10.3) |
| **Physical** | 87.5 (10.7) | 77.9 (12.6) | 88.9 (10.8) | 90.3 (10.1) | 90.0 (10.8) | 91.6 (8.1) |

**Note.** Descriptive statistics only, no inferential tests are presented.

PedsQL= Pediatric Quality of Life Inventory. Adapted from Varni, Seid, and Kurtin (2001) [9].

¹Psychosocial Summary Score = mean of Emotional, Social, and School Functioning (PedsQL).
